# A review of the validity of computerized neurocognitive assessment tools in mild traumatic brain injury assessment

**DOI:** 10.2217/cnc-2016-0021

**Published:** 2017-01-30

**Authors:** Jacques P Arrieux, Wesley R Cole, Angelica P Ahrens

**Affiliations:** 1 Womack Army Medical Center, Fort Bragg, NC, USA; 2 Defense & Veterans Brain Injury Center (DVBIC), Fort Bragg, NC, USA

**Keywords:** computerized cognitive testing, computerized neurocognitive assessment tools, computerized neurocognitive test, literature review, psychometric, neuropsychological assessment, validity

## Abstract

Computerized neurocognitive assessment tools (NCATs) offer potential advantages over traditional neuropsychological tests in postconcussion assessments. However, their psychometric properties and clinical utility are still questionable. The body of research regarding the validity and clinical utility of NCATs suggests some support for aspects of validity (e.g., convergent validity) and some ability to distinguish between concussed individuals and controls, though there are still questions regarding the validity of these tests and their clinical utility, especially outside of the acute injury timeframe. In this paper, we provide a comprehensive summary of the existing validity literature for four commonly used and studied NCATs (automated neuropsychological assessment metrics, CNS vital signs, cogstate and immediate post-concussion and cognitive testing) and lay the groundwork for future investigations.

The measurement of cognitive functioning via neuropsychological (NP) testing is an important component of assessment after mild traumatic brain injury (mTBI), also known as concussion. A consensus statement on concussion in sport [1] concluded that such testing provides valuable information when evaluating a person with mTBI. The US military also mandates that service members are administered NP assessment to detect cognitive impairment associated with mTBI [2].

Traditional NP assessments are typically comprised of well-established measures with large normative databases and demonstrate evidence of adequate psychometric properties (i.e., reliability and validity). However, these tests are usually administered in a one-on-one format by a trained professional with paper, pencil and stopwatch, and require interpretation by a neuropsychologist. This can make them expensive and time intensive, not feasible for assessing large groups (e.g., athletic teams, service members) or using on the sideline or in combat settings. Over the past few decades, alternatives to traditional NP assessment batteries have emerged in the form of computerized neurocognitive assessment tools (NCATs).

NCATs offer several potential logistical advantages over traditional NP tests. They can be much less time consuming and do not require administration by a testing specialist. Scoring is automated, and test performance can be easily generated into a summary report for interpretation or an electronic spreadsheet for statistical analysis. Furthermore, NCATs allow for cognitive assessment to be obtained in geographic areas where traditional NP services are limited. They are easier to use for obtaining baseline tests (e.g., preseason, predeployment) that can be used for comparison to assessment after concussion, which can be especially advantageous where examinees may have conditions that prevent comparison to normative reference groups (i.e., abnormal cognitive development, ADHD, among others) [3,4]. Also, the computerized nature of NCATs makes it possible to administer alternative forms of a test with numerous combinations of test stimuli, which mitigate practice effects, and allow for multiple administrations in a short amount of time to track recovery after injury [3]. Moreover, being computerized allows for more accurate measurement of reaction time (RT), possibly making NCATs more sensitive to subtle cognitive effects [5].

Despite potential advantages, NCATs are not without limitations, as discussed by Echemendia *et al*. [4]. Specifically, alternate forms might not be equivalent, computers settings can cause erroneous RT measurement, there are differences between administering to groups (as is often done for baseline administrations) and to individuals (as is often done postinjury), and the tests are marketed to professionals (e.g., athletic trainers) who may have little or no training in cognitive testing. Additionally, one of the most important limitations of NCATs is that the psychometric properties are not fully established. Although NCATs have gained momentum as a tool in the management of mTBI, particularly in military and athletic settings, commonly used NCATs have not undergone the same level of validation as many traditional tests. According to a Joint Position Paper of the American Academy of Clinical Neuropsychology and the National Academy of Neuropsychology [3], though NCATs may seem to be analogous to traditional NP tests, there are important differences between them that need to be explored. Specifically, modifications of existing measures warrant investigations of the new tests’ psychometric properties, such as validity [6].

This manuscript will summarize and evaluate the existing literature regarding the validity of four NCATs commonly used for both clinical and research purposes: Automated Neuropsychological Assessment Metric (ANAM), CNS-Vital Signs (CNS-VS), Axon/CogState/CogSport (CogState) and Immediate Post-Concussion Assessment and Cognitive Testing (ImPACT; please see Table 1 for a description of each NCAT). The tests that will be covered are commonly used in research and clinical settings. Specifically, CNS-VS has been used in several clinical trials and can be billed to medical insurance, ANAM is required for US military Service Members, CogState is commonly used in Australian athletic settings and ImPACT has been approved by the US FDA to detect cognitive deficits following mTBI. Interestingly, to date, these measures have not generated adequate evidence of validity, yet they are commonly used for TBI-related assessment in sports and military settings. This summary and review will focus on the comparisons of these tests to traditional NP batteries as well as evaluations of the ability of these tests to provide clinically meaningful information regarding cognitive functioning after concussion. The existing state of the literature will be evaluated based on criteria put forth by Randolph *et al*. in a 2005 [7] review of the literature regarding NP testing after sport-related concussion (SRC). That review and those criteria are discussed below (see Box 1). This is not a systematic literature review, but is rather meant to serve as a concise summary and reference, with recommendations for future studies and considerations identified.

**Table 1.** **Descriptions of computerized neurocognitive assessment tools reviewed.**

**Test**	**Subtests and cognitive construct intended to measure**	**Classification and summary scores**
ANAM4	Simple Reaction Time (SRT): visuomotor processing speed, simple motor speed and attention Procedural Reaction Time (PRO): processing speed, visuomotor reaction time and attention Code Substitution Learning (CDS): visual scanning, visual perception, attention, associative learning and processing speed Code Substitution Delayed (CDD): learning and delayed visual recognition memory Mathematical Processing (MTH): basic computational skills, concentration and working memory Matching to Sample (M2S): visual-spatial processing, working memory and visual recognition memory SRT, Second Administration (SRT2): index of attention (i.e., reaction time [RT] and vigilance)	Summary scores: Throughput (TP; number of correct responses per minute of available response time) Standardized subtest TP – standardized composite TP Composite score (standardized average TP [*z*-score]) Classification of impairment: ANAM composite *z* ≤ -1.28

CNS-VS	Verbal Memory Test, Immediate (VBM): word recognition and memory, immediate and delayed recall Visual Memory Test, Immediate (VIM): visual recognition and memory, immediate and delayed recall Finger Tapping Test (FTT): motor speed, fine motor control Symbol Digit Coding (SDC): information processing speed, complex attention, visual perceptual speed Stroop Test (ST): SRT, complex reaction time, inhibition, executive skills, processing speed Shifting Attention Test (SAT): executive functioning, RT Continuous Performance Test (CPT): sustained attention, choice reaction time (CRT), impulsivity Verbal Memory Test, Delayed (VBM): word recognition, memory and delayed recall Visual Memory Test, Delayed (VIM): visual recognition, memory and delayed recall	Summary scores: Neurocognitive Index (NCI) Composite memory Verbal memory Visual memory Psychomotor speed RT Complex attention Cognitive flexibility Processing speed Executive function Simple attention Motor speed Composite score: IQ scale and percentile Classification of impairment: N/A

CogState	Detection Task: SRT Identification Task: processing speed One Back Task: attention, working memory One Card Learning Task: learning and recognition memory	Summary scores: score for each subtest Composite score: *z*-scores Classification of impairment: -1.64 SD on at least two subtests CogState composite < -1.64

ImPACT	Word Memory, Immediate: verbal recognition memory Design Memory, Immediate: visual recognition memory X's and O's: visual working memory and visual processing/visual motor speed Symbol Match: visual processing speed, learning and memory Color Match: CRT and impulse control/response inhibition Four Letters: working memory and visual-motor response speed Word Memory, Delayed: verbal recognition memory Design Memory, Delayed: visual recognition memory	Summary scores: Verbal memory Visual memory Visual motor speed RT Impulse control Composite score: Test-specific Standardized scores and percentiles Classification of impairment: N/A

Note: Each NCAT is online or desktop based and the approximate administration time, inclusive of testing and acquisition of medical history and demographics, is 30 min, with the exception of CogState (20 min). These summaries generally capture the ‘standard’ battery for each NCAT; however, the batteries used in the reviewed studies may include some variations and different combinations of subtests.

ANAM4: Automated neurocognitive assessment metric, version 4; CNS-VS: CNS-vital sign; ImPACT: Immediate post-concussion assessment and cognitive testing; IQ: Intelligence quotient; N/A: Not applicable; NCAT: Neurocognitive assessment tool.

Adapted with permission from [8].

## Validity

Prior to discussing the literature on validity, it is important to establish what is meant by the term ‘validity’ (see Box 2). Validity is the most important aspect of test construction and thus is a key consideration when evaluating the clinical utility of a test. In psychometric research, validity describes whether a test measures what it claims to measure, by meeting the criteria that have been established to determine its accuracy [6]. Various models of test validity have been proposed [9,10], though in general, there are three ways to describe the validity of a test: by its content, by the construct it is purported to measure or by its ability to measure a certain criterion [11,12].

Content validity describes the relevance of the test items to the construct that is to be measured [11,12]. For example, determining if a test of attention is comprised of test items and stimuli that accurately and adequately measure attention, rather than some other construct, such as RT. Content validity is often assessed by the subjective agreement among subject matter experts, such as neuropsychologists, that the test items are relevant and appropriate for the test purpose. Construct validity describes the extent to which the measure represents the basic theoretical construct, such as cognitive functioning. It is primarily evaluated with correlations, regression or factor analysis between a domain of interest and other well-established, ‘gold-standard’ measures [11–13]. It is typically conceptualized as convergent and discriminant validity. Specifically, tests assessing similar constructs should have higher correlations (i.e., convergent validity) than tests of dissimilar constructs (i.e., discriminant validity). Criterion validity describes the relatedness of the measure to a specified criterion, such as a condition of interest or outcome (e.g., concussion), and is often divided into concurrent and predictive types of validity. Concurrent validity is determined by how well a test accurately identifies a diagnosis or condition of interest when that condition is known (e.g., control vs concussion cohorts), as compared with an existing ‘gold standard’. Predictive validity is determined by the test's ability to inform about some type of future outcome. It is important to note that there can be some overlap in these different types of validity, as they are not meant to conceptually represent mutually exclusive subcategories of validity, but rather describe the various ways in which validity can manifest [12].

## Past literature reviews

There are several existing literature reviews of NCATs, including those focused on one NCAT, such as ANAM or ImPACT, for example, [14,15], as well as those focused on the broader body of NCAT literature, for example, [7–8,16]. In a comprehensive review of literature on NP testing (traditional and computerized) in SRC published from 1990 to 2004, Randolph *et al*. [7] identified several gaps with regard to the use of both traditional and computerized NP testing after SRC. They proposed five criteria that needed to be satisfied with additional research in order to consider NP testing standard of care after concussion (see Table 1). Until these requirements are satisfied, the authors suggest that professionals should use NP tests, including NCATs, with caution and rely more on self-report measures and medical evaluations. In this literature review, we will place a specific focus on the validity-related ‘Randolph criteria’ (i.e., criteria two through five) in order to establish whether the existing research, including the research that has emerged since their review, sufficiently demonstrates that NCATs have satisfied those criteria and demonstrate adequate clinical utility.

Reviews since the Randolph *et al*. [7] paper seem to indicate that while the Randolph criteria have been partially addressed, there is still insufficient evidence that NCATs adequately satisfy the criteria. Resch *et al*. [8] conducted a similar literature review as Randolph *et al*. [7], though for research completed between 2005 and 2013, and for NCATs used primarily for SRC. The authors reported that the evidence of validity varies between NCATs, suggesting that more research is necessary in order to elucidate the relationship between NCATS and their traditional NP counterparts. Iverson and Schatz [16] conducted a literature review of NP assessment in SRC research and presented some evidence indicating that NCATs may be superior to their traditional counterparts because they can be more precise in the detection of cognitive impairment. However, all subsequent reviews, similar to Randolph *et al*.'s [7] conclusions, suggest additional research is needed in order to further validate NCATs against their traditional NP counterparts and within mTBI populations.

### Summary of literature

The sections below provide a review of the literature published to date investigating the validity of the four NCATS: ANAM, CNS-VS, CogState and ImPACT. Their utility as a neurocognitive assessment is presented in two contexts: the extent to which the test measures the same constructs as traditional NP batteries and the extent to which it provides clinically meaningful information about group membership or cognitive impairment. The reader should refer to Tables 2–5 for details on the specifics of the methodology and findings of each of the studies described (as well as the full definitions of NP test-specific acronyms).

**Table 2.** **Summary of Automated Neuropsychological Assessment Metric validity studies.**

**Study (year)**	**Study sample**	**Methods: tests**	**Methods: statistics**	**Results**	**Ref.**
Bleiberg *et al*. (2000)	122 healthy high school and college students	Compared ANAM to WAIS-R, Finger Tapping, TMT A and B, CT total, PASAT, HVLT and Stroop Color-Word Test	Pearson *r* correlation coefficients Stepwise regression PCA	Correlations ranged from -0.60 to 0.66 Stepwise regression: MTH and STN predict NP test scores PCA: identified a four-factor solution accounting for 66% of the variance	[17]

Kabat *et al*. (2001)	191 veterans referred for outpatient NP testing	Compared ANAM to WAIS-R Digit Symbol Coding, CVLT and TMT A and B	Pearson *r* correlation coefficients Stepwise regression PCA	Correlations ranged from -0.64 to 0.66. CDS best predict TMT B and WAIS-R DS PCA identified two three-factor solutions accounting for 60–62% variance	[18]

Woodard *et al*. (2002)	Uninjured high school athletes	Compared ANAM to HVLT, COWAT, WAIS-III DS and SS, BTA	Pearson *r* correlation coefficients	Correlations ranged from -0.07 to 0.82. MTH most associated with traditional NP test scores MTH was significantly correlated with four of six traditional scores	[19]

Jones *et al*. (2008)	77 healthy college students	Compared ANAM to WJ-III	Pearson *r* correlation coefficients Stepwise regression PCA	Correlations ranged from -0.10 to 0.55. Strongest correlation was between LGR TP and GIA scores LGR and MTH best predictors of WJ-III (Efficiency Index and Visual Matching) PCA: MTH identified as a distinct factor, accounting for 19% variance	[20]

Woodhouse *et al*. (2013)	143 patients referred for outpatient NP assessments. RBANS used to group participants as impaired (n = 30) and not impaired (113)	Compared ANAM to RBANS	RBANS Total Index score (≤15th percentile) Pearson *r* correlation coefficients Logistic regression	Correlations ranged from 0.01 to 0.52. Strongest correlation was between MTH and RBANS total scores ANAM Composite TP score accounted for 37% variability in RBNAS Total Index Score ANAM subtest scores on predicting RBANS impairment: sensitivity = 81%, specificity = 89.1%, PPV = 56.7%, NPV = 87.9%, odds ratio = 34.65	[21]

Bleiberg and Warden (2002)	US Military Academy cadets, 68 with mTBI and 16 healthy controls	Administered ANAM at baseline and then again four-times over 2-week period (first 2 weeks of recovery for mTBI group)	RCI-defined impairment Fisher's exact test	mTBI group, RCI-based decline in 19% (2 scores) and 81% (1 score) Fisher's: MTH identified 21% of mTBI group. Fisher's: mTBI failed to demonstrate practice effect (80%)	[22]

Bleiberg *et al*. (1997)	Six with mTBI and six healthy controls	Administered ANAM and WAIS-R, WRAT-R, FFT, Stroop Color-Word Interference, PASAT, CVLT and COWAT ANAM administered 30-times in four sessions over a 2-week period	MANOVA	MANOVA: Group differences in three out of five of the ANAM subtests Practice effects in control group, while mTBI group's improvement was shorter and more variable	[23]

Kelly *et al*. (2012)	71 with acute mTBI and 166 controls, deployed in combat environments	Administered ANAM, traditional NP battery and questionnaires within 72 h of injury	ROC curve, including AUC	Difference at enrollment: SRT + PRO, AUC = 0.73, discriminant ability = 71%<br> sensitivity = 59%, specificity = 82% Change from baseline: SRT + MTH + M2S, AUC = 0.79 Discriminant ability = 75%, sensitivity = 53%, specificity = 98%	[24]

Coldren *et al*. (2012)	47 with mTBI and 108 healthy controls, deployed in combat environments	Compared performance at predeployment baseline and ≤3, 5, 10+ days postinjury	Mann-Whitney U	Significant differences on five of six ANAM subtests at ≤3-day postinjury No differences at 5 and 10+ days intervals.	[25]

Norris *et al*. (2013)	165 soldiers with mTBI	Correlated performance on ANAM with demographic variables (age, number of blast exposures, % of prior mTBI) at 3- and 5-day postinjury	Spearman's *ρ* statistics *z*-scores Kaplan–Meier plot	Spearman's *ρ*: day 5 ANAM performance was associated with Return to Duty (RTD) rather than demographics SRT2 at day 3 most correlated with RTD time Kaplan-Meier: lowest 25% took 19 days RTD, highest 25% took 7 days RTD	[26]

Register-Milhalik *et al*. (2013)	38 healthy college players and 132 college athletes with mTBI	Compared ANAM and SOT and GSC. Control group was tested two-times (average of 4 days apart). mTBI group was tested two-times (preseason baseline and within 5 days following mTBI)	RCI-defined impairment sensitivity and specificity	ANAM (80% CI, average of seven subtests): sensitivity = 0.09, specificity = 0.95 When combined with SOT and GSC, ANAM (80% CI): sensitivity = 0.50, specificity = 0.96	[27]

Nelson *et al*. (2016)	165 mTBI and 166 healthy controls, athletes	Administered ANAM at baseline and then at 1, 8, 15 and 45 days (postinjury/baseline)	ANOVA Cohen's *d* effect sizes RCI-defined impairment ROC curve, including AUC	ANOVA: significant for 7 of 9 scores at day 1 (*d* ranged from -0.58 to -0.84). Limited significant differences at days 8, 15 and 45. AUC: significant for 7 of 9 scores at 1-day assessment (0.63–0.73). Limited significant differences at days 8, 15 and 45	[28]

ANAM: Automated neuropsychological assessment metric; ANOVA: Analysis of variance; AUC: Area under the curve; BTA: Brief Test of Attention; CDS: Code substitution learning; COWAT: Controlled Oral Word Association Test; CT: Consonant Trigrams; CVLT: California Verbal Learning Test; DS: Digit Symbol; FFT: Finger Tapping Test; GIA: General intellectual ability index; GSC: Graded Symptom Checklist; HVLT: Hopkins Verbal Learning Test; LGR: Logical reasoning; M2S: Matching to sample; MANOVA: Multiple analysis of variance; mTBI: Mild traumatic brain injury; MTH: Mathematical processing; NP: Neuropsychological; NPV: Negative predictive value; PASAT: Paced Auditory Serial Addition Test; PCA: Principal component analysis; PPV: Positive predictive value; PRO: Procedural reaction time; RBANS: Repeatable Battery For the Assessment of Neuropsychological Status; RCI: Reliable Change Index; ROC: Receiver operating characteristics; RTD: Return to Duty; SOT: Sensory Organization Test; SRT: Simple reaction time; SS: Symbol Search; STN: Sternberg memory procedure; TMT: Trail Making Test; WAIS-III Wechsler Adult Intelligence Scale-Third Edition; WAIS-R: Wechsler Adult Intelligence Scale-Revised; WJ-III: Woodcock Johnson, Test of Cognitive Abilities-Third Edition; WRAT-R: Wide Range Achievement Test-Revised.

**Table 3.** **Summary of CNS-Vital Signs validity studies.**

**Study (year)**	**Study sample**	**Methods: tests**	**Methods: statistics**	**Results**	**Ref.**
Gualtieri and Johnson (2006)	144 with neuropsychiatric disorders and 36 healthy controls	Compared CNS-VS to RAVLT, WMS LM subtests, FTT, the ST, TMT B and the VF test	Pearson *r* correlation coefficients	Correlations ranged from -0.53 to 0.79 Strongest correlation was between CNS-VS SDC and WAIS DS.	[29]

Lanting *et al*. (2012)	50 with mTBI	Compared CNS-VS to NAB, RST, WTAR. Assessed at 6–8 weeks following injury	Pearson *r* correlation coefficients	Correlations ranged from 0.28 to 0.58 Strongest correlation was between CNS-VS Psychomotor Speed score and NAB memory index standard score (*r* = 0.58)	[30]

Gualtieri and Hervey (2015)	179 with psychiatric disorders	Compared CNS-VS to WAIS-III	Pearson *r* correlation coefficients Exploratory and confirmatory factor analyses Stepwise discriminant function analysis Logistic regression	Correlations ranged from 0.33 to 0.59. Strongest correlation was between CNS-VS SAT and FSIQ CNS-VS SAT and VIM scores were the only significant predictors of FSIQ	[31]

Lanting *et al*. (2012)	50 with mTBI and 31 with orthopedic injury	Administered CNS-VS at 6–8-week postinjury	MANOVA	No significant differences between groups	[32]

Gualtieri and Johnson (2008)	145 controls and 141 examinees with TBI separated into four groups: PCS = 13; mTBI recovered = 15; recovered from STBI = 85, unrecovered STBI = 28	Administered CNS-VS. PCS tested within 3-month postinjury mTBI tested within 12-month postinjury STBI time since injury unspecified	MANOVA *Post hoc t*-tests comparing the five groups ROC curve, including AUC	MANOVA: 18 of 28 scores were significantly different. *Post hoct*-tests demonstrated significant differences on most CNS-VS scores between healthy controls and the four TBI cohorts, with mTBI recovered performing as well as healthy controls *Post hoct*-tests demonstrated the STBI groups performed significantly worse than the mTBI groups AUC, group membership between injured and healthy groups: significant for psychomotor speed (0.75), NCI (0.75) and cognitive flexibility (0.71)	[33]

Dresch *et al*. (2015)	458 active-duty soldiers (deemed fit for duty)	Administered CNS-VS, demographic questionnaires and biomarkers 30 days before and after deployment	Cohen's *d* effect sizes	Cohen's *d* = 0.22 on pre–post-deployment comparisons Cohen's *d* = 0.40 on postdeployment comparisons, with the sample divided into ‘no traumatic stress’ or ‘traumatic stress groups’	[34]

AUC: Area under the curve; CNS-VS: CNS-vital sign; FSIQ: Full Scale Intelligence Quotient; FTT: Finer Tapping Test; LM: Logical memory; MANOVA: Multivariate analysis of variance; mTBI: Mild traumatic brain injury; NAB: Neuropsychological assessment battery; NCI: Neurocognitive Index; PCS: Postconcussive syndrome; RAVLT: Rey Auditory Verbal Learning Test; ROC: Receiver operating characteristics; RST: Reynolds Intellectual Screening Test; SAT: Shifting attention test; SDC: Symbol digit coding; ST: Stroop Test; STBI: Severe TBI; TMT: Trail Making Test; VIM: Visual memory test; WAIS DS: Wechsler adult intelligence scale digit symbol; VF: Verbal fluency; WAIS-III: Wechsler Adult Intelligence Scale-Third Edition; WMS: Wechsler Memory Test; WTAR: Wechsler Test of Adult Reading.

**Table 4.** **Summary of Axon/CogState/CogSport validity studies.**

**Study (year)**	**Study sample**	**Methods: tests**	**Methods: statistics**	**Results**	**Ref.**
Makdissi *et al*. (2001)	240 healthy athletes. Six of original cohort sustaining acute mTBI and seven matched controls were retested	Compared CogState's SRT subtest to DSST and TMT B mTBI group tested at baseline and then at 72-h postinjury Control group retested ∼34 days following baseline	Pearson *r* correlation coefficients for each group MANOVA	Correlations: Better for controls than mTBI SRT and DSST: *r* = -0.48.x SRT and TMT: *r* = 0.42 MANOVA: mTBI group had significant (36%) decline in performance on CogState SRT RT variability was significantly different with the mTBI group	[35]

Collie *et al*. (2003)	240 elite athletes and 60 demographically matched controls	Compared CogState to DSST and TMT-B	Pearson *r* correlation coefficients	Correlations ranged from -0.86 to 0.44 Strongest correlation was between CogState Decision-Making Speed and DSST	[36]

Schatz and Putz (2006)	30 healthy controls	Compared CogSport to TMT and WAIS-R DS. Also administered ImPACT Administered three-times over a 5-day period, at 48-h intervals	Pearson *r* correlation coefficients	Correlations ranged from -0.28 to 0.54 Strongest correlation was between CRT and TMT A and B	[37]

Maruff *et al*. (2009)	215 healthy controls 50 participants with cognitive impairment (mTBI, schizophrenia, AIDS Dementia Complex)	Compared CogState to GPB, TMT, SDMT, BVMT, RCFT and WMS-III SS subtest	Pearson *r* correlation coefficients ANOVA Cohen's *d* effect sizes Non-OL%	Correlations ranged from 0.49 to 0.83 ANOVA: significant differences and medium to large effect sizes for impairment (0.60 to -1.80) Non-OL%: CogState subtests could identify 53–78% impairment that was unique to mTBI alone	[38]

Collie *et al*. (2006)	615 Australian Rules football players completed baseline assessments 61 sustained mTBI and 84 healthy controls were retested	Compared CogState to TMT-B, DSST and standardized symptom checklist. Tests administered at baseline retested 11 days later. Symptom assessment used to categorize examinees as symptomatic or asymptomatic	ANOVA *z*-score change	Control group, no significant changes from baseline Asymptomatic significantly declined on one of seven scores Symptomatic group significantly declined on three of seven scores (z-score change ranged from -0.60 to -0.86)	[39]

Louey *et al*. (2014)	29 athletes with acute mTBI and 260 healthy control athletes	Administered CogState Compared normative and baseline comparison methods to classify cognitive impairment (subtest scores ≤1.65 SD below mean)	Reliable Change Index (RCI)-defined impairment ANCOVA Cohen's *d* effect sizes Chi-squared analyses to determine diagnostic accuracy of impaired performance CCR	ANCOVA: significant differences between groups on all scores Cohen's *d* ranged from -0.94 to -2.95 Chi-square: baseline data, sensitivity = 96.6%, specificity = 86.9%, CCR = 87.9% Normative data, sensitivity = 69%, specificity = 91.5%, CCR = 89.3%	[40]

Gardner *et al*. (2012)	51 rugby players with acute mTBI and 41 controls	Compared CogState/CogSport to WAIS-III. Also administered ImPACT	*t*-tests Effect sizes Logistic regression	Significant differences between groups on four out of five CogState tests Effect sizes ranged from -0.46 to -0.88 Logistic regression: CogState improved classification accuracy 3.5% beyond demographics	[41]

Nelson *et al*. (2016)	165 athletes with mTBI and 166 healthy controls	Administered CogState at baseline and then at 1, 8, 15 and 45 days (postinjury/baseline)	ANOVA Cohen's *d* effect sizes RCI-defined impairment ROC curve, including AUC	ANOVA: significant differences for four of five scores at day 1 (*d* ranged from -0.51 to -0.72). Limited significant differences at days 8, 15 and 45 AUC: significant for four of five scores at day 1 (0.64–0.69). No significant differences at days 8, 15 and 45	[28]

AUC: Area under the curve; ANCOVA: Analysis of covariance; ANOVA: Analysis of variance; BVMT: Brief Visual Memory Test; CCR: Correct Classification Rate; CRT: Choice reaction time; DS: Digit Symbol; DSST: Digit Symbol Substitution Test; GPB: Grooved Pegboard; ImPACT: Immediate post-concussion assessment and cognitive testing; MANOVA: Multivariate analysis of variance; mTBI: Mild traumatic brain injury; Non-OL%: Nonoverlap statistics; RCFT: Rey Complex Figure Test; RCI: Reliable Change Index; ROC: Receiver operating characteristics; RT: Reaction time; SDMT: Symbol Digit Modalities Test; SRT: Simple Reaction Time; SS: Spatial span; TMT: Trail Making Test; WAIS-III: Wechsler Adult Intelligence Scale-Third Edition; WAIS-R: Wechsler Adult Intelligence Scale-Revised; WMS-III: Wechsler Memory Scale-Third Edition.

**Table 5.** **Summary of Immediate Postconcussion Assessment and Cognitive Testing validity studies.**

**Study (year)**	**Study sample**	**Methods: tests**	**Methods: statistics**	**Results**	**Ref.**
Iverson *et al*. (2005)	72 athletes with acute mTBI.	Compared ImPACT to SDMT	Pearson *r* correlation coefficients EFA	Correlations ranged from -0.60 to 0.70 EFA: revealed a two-factor solution describing, first, speed/RT and second, memory	[42]

Schatz and Putz (2006)	30 healthy controls	Compared ImPACT to TMT and WAIS-R DS. Also administered CogState Administered three-times over a 5-day period, at 48-h intervals	Pearson *r* correlation coefficients	Correlations ranged from -0.506 to 0.641 Strongest correlation was between CRT and TMT-A (0.54) and TMT-B (0.54)	[37]

Maerlender *et al*. (2010)	68 healthy controls	Compared ImPACT to CVLT, BVMT-R, DKEFS, CPT, GPB and PASAT	Pearson *r* correlation coefficients Canonical correlations	Correlations ranged from -0.39 to 0.59 Canonical correlations: two of the five canonical dimensions were significant (0.80 and 0.73), indicating the batteries measured similar constructs	[43]

Maerlender *et al*. (2013)	68 healthy controls	Conducted additional analyses of Maerlender *et al*. (2010) data	Point-biserial correlations	Point-biserial correlation: ImPACT did not significantly discriminate between dissimilar measures	[44]

Allen and Gfeller (2011)	100 healthy controls	Compared ImPACT to the NFL NP battery: HVLT-R, BVMT-R, TMT, COWAT and WAIS-III	Pearson *r* correlation coefficients	Correlations ranged from -0.38 to 0.43. Strongest correlation was between WAIS-III DS and ImPACT Visual Motor Speed	[45]

Solomon and Kuhn (2014)	226 NFL draft picks, with and without mTBI history	Compared ImPACT to Wonderlic	Pearson *r* correlation coefficients	Correlations ranged from -0.26 to 0.49. Strongest correlation was with Visual Motor Speed	[46]

Van Kampen *et al*. (2006)	122 athletes with acute mTBI and 70 healthy controls	Administered ImPACT at baseline and then at 2-day postinjury for mTBI group, and postseason for controls	RCI-defined impairment Percent change from baseline	93% of the mTBI group performed lower than at their baseline 30% of control group performed lower than baseline	[47]

Broglio *et al*. (2007)	75 with mTBI high school students	Compared ImPACT to HVLT, TMT, SDMT, DS, COWAT	Chi-square	ImPACT demonstrated better sensitivity to mTBI (62.5%) than traditional battery (43.5%)	[48]

Covassin *et al*. (2008)	57 athletes with mTBI (36 with no history of mTBI, 21 with history of 2+ mTBI)	Administered ImPACT at baseline and then at 1- and 5-day postinjury	Chi-square and univariate *post hoc* tests	mTBI group exhibited significantly lower scores on Verbal Memory (p = 0.01) and RT (p = 0.023) at 5-day postinjury	[49]

Gardner *et al*. (2012)	51 rugby players with acute mTBI and 41 controls	Compared ImPACT to WAIS-III. Also administered CogState	ANOVA Cohen's *d* effect sizes Logistic regression to distinguish between groups	Significant differences between groups on 1/4 of the ImPACT scores. Cohen's *d* effect size = -0.57 Logistic Regression: ImPACT improved classification accuracy 3.5% beyond demographics	[41]

Schatz *et al*. (2006)	72 high school athletes with mTBI and 66 healthy controls with no history of mTBI	Administered ImPACT at 3-day postinjury	MANOVA DFA: classification of group status	MANOVA: significant differences between groups on four out of five index scores (partial Eta^2^ ranged from 0.19 to 0.31). DFA: sensitivity = 81.9, specificity = 89.4	[50]

Schatz and Maerlender (2013)	Analyzed pre-existing data of 21,537 athletes’ baseline assessments and 560 post-mTBI	Performed factor analysis on preexisting ImPACT datasets	Factor analysis	Identified two primary cognitive factors: first, memory (verbal and visual memory) and second, speed (visual motor speed and RT)	[51]

Schatz and Sandel (2013)	Analyzed pre-existing data of athletes with mTBI (81 symptomatic and 37 asymptomatic), and matched controls (n's = 81 & 37)	Administered ImPACT at baseline and then at 3-day postinjury for mTBI group, and postseason for controls	DFA	DFA: symptomatic vs controls Symptomatic group: sensitivity = 91.4%, specificity = 69.1% DFA: asymptomatic vs controls: sensitivity = 94.6%, specificity = 97.3%	[52]

Nelson *et al*. (2016)	165 athletes with mTBI and 166 healthy controls	Administered ImPACT at baseline and then at 1, 8, 15 and 45 days (postinjury/baseline)	ANOVA Cohen's *d* effect sizes RCI-defined impairment ROC curve, including AUC	ANOVA: significant for four or five scores at day 1 (Cohen's *d* ranged from -0.70 to -0.80). Minimal significant differences at days 8, 15, or 45 AUC: significant for four of five scores at day 1 (0.70–0.71). Minimal significant differences at days 8, 15 and 45	[28]

AUC: Area under the curve; ANOVA: Analysis of variance; BVMT-R: Brief Visual Memory Test-Revised; COWAT: Controlled Oral Word Association Test; CPT: Continuous Performance Test; CRT: Choice Reaction time; CVLT: California Verbal Learning Test; DFA: Discriminant Functional Analysis; DKEFS: Delis Kaplan Executive Functioning System; DS: Digit Symbol; EFA: Exploratory factor analysis; GPB: Grooved Pegboard; HVLT-R: Hopkins Verbal Learning Test-Revised; ImPACT: Immediate postconcussion assessment and cognitive testing; MANOVA: Multivariate analysis of variance; mTBI: Mild traumatic brain injury; NFL: National Football League; NP: Neuropsychological; PASAT: Paced Auditory Serial Addition Test; RCI: Reliable Change Index; ROC: Receiver operating characteristics; RT: Reaction time; SDMT: Symbol Digit Modalities Test; TMT: Trail Making Test; WAIS-III: Wechsler Adult Intelligence Scale-Third Edition; WAIS-R: Wechsler Adult Intelligence Scale-Revised.

## Methods

The search for primary literature involved several search engines (e.g., Google Scholar, PubMed, EBSCOhost and ScienceDirect). Articles were chosen based on their relevance to evaluations of the validity of the four above-mentioned NCATs. Specifically, the selection criteria were based on search terms such as ANAM, CNS-VS, CogState/CogSport/Axon and ImPACT in conjunction with any number of the following terms: validity, validation, construct validity, criterion validity, convergent validity, discriminant validity, diagnosis, group differences, sensitivity/specificity, mTBI and concussion. Studies were primarily included if analyses involved either, first, comparison of performance on NCATs and traditional NP tests or second, comparison of group differences in NCAT performance between healthy controls and individuals who sustained an mTBI. Revisions to this methodology (i.e., extending relevant study populations to those with neuropsychiatric disorders) were permitted as alternative ways of capturing measures of NCAT validity when search findings were insufficient. For example, we included several articles that studied adolescent samples as many studies of SRC combined high school and college athletes. In addition, several studies of non-mTBI samples (e.g., psychiatric disorders) were also included as they often compared NCAT scores to traditional NP tests in a group of healthy controls. Since this was not a rigorous and systematic literature review, the conclusions drawn should be considered with caution by the reader. However, we believe consolidating these findings in a single review that is invaluable for those interested in knowing where the literature currently stands in regards to the validity and clinical utility of these NCATs.

## Automated neuropsychological assessment metric

### Comparisons to traditional NP tests

Research to date has largely demonstrated that scores on ANAM and traditional NP tests have weak-to-moderate correlations (see Table 2 for more details on the methodology and findings of these studies). Bleiberg *et al*. [17] concluded that ANAM measures similar cognitive constructs as traditional NP batteries in a group of healthy controls, as correlations were generally moderate. ANAM throughput (TP) scores more strongly correlated with traditional NP test scores than RT and accuracy, and ANAM Mathematical Processing (MTH) and Sternberg Memory Procedure (STN) were most closely associated with scores from the Paced Auditory Serial Addition Test in the traditional NP battery. Kabat *et al*. [18] similarly found moderate correlations in a group of veterans, the strongest of which were between the ANAM Code Substitution Learning median RT and Trail Making Test (TMT) B. However, the median RT score is not a commonly used ANAM score for clinical or research purposes. In another study, with uninjured high school athletes, MTH demonstrated the most statistically significant correlations (i.e., moderate to strong) with a traditional NP score (Digit Symbol Coding) [19]. In a comparison of healthy college students’ performance on ANAM and Woodcock Johnson, Test of Cognitive Abilities-Third Edition (WJ-III), Jones *et al*. [20] found some evidence of construct validity, as ANAM moderately correlated with many of the WJ-III subtests and clusters, with the strongest correlation between the WJ-III General Intellectual Ability index (GIA) score and the ANAM Logical Reasoning (LGR) TP score. Woodhouse *et al*. [21] additionally observed several statistically significant correlations between the ANAM and Repeatable Battery for the Assessment of Neuropsychological Status (RBANS), administered to a mixed clinical sample referred for assessments of cognitive functioning. These patients were diagnosed postevaluation with a variety of neurologic and psychiatric disorders. Each of the seven ANAM subtests was correlated with RBANS performance. The strongest correlation existed between ANAM MTH TP and the RBANS Total Index score.

Studies using regression analyses investigate the ability of ANAM to predict scores on traditional NP batteries. Results have generally provided evidence for construct validity, as certain ANAM scores can significantly predict performance on traditional NP batteries [17–18,20–21]. MTH, STN and LGR appear to be the ANAM subtests that best predict performance on traditional tests such as Wechsler Adult Intelligence Scale-Revised (WAIS-R), WJ-III, TMT and RBANS. Principal component analysis (PCA) has also been used to further investigate the relationship between scores on ANAM and traditional NP tests. Generally, data from such studies also provide evidence for construct validity, demonstrating that ANAM is assessing underlying cognitive constructs of efficiency, working memory and resistance to interference similarly to traditional NP tests [17–18,20].

### Group differences

Results from several studies to date suggest that ANAM may have some diagnostic utility in mTBI cases, particularly in the acute phase of injury (see Table 2 for accompanying details on the existing literature to supplement this text). Bleiberg and Warden [22] administered baseline ANAM assessments to US Military Academy cadets and made comparisons in performance at four time points over a 2-week period (during the first 2 weeks after injury for those in the mTBI cohort). Using ANAM's Reliable Change Index (RCI) as a cutoff for impairment [53,54]), ANAM scores were generally able to differentiate examinees with mTBI from healthy controls, as the mTBI group had two or more RCI-based performance declines, while controls did not. Additionally, significant practice effects were only demonstrated in the control group (53%). The authors suggested that the absence of a practice effect in the mTBI group might be one of the better ways to identify cognitive impairment following concussion.

An earlier study [23] investigating the diagnostic capabilities of ANAM as compared with traditional NP batteries found differences between the mTBI and demographically matched control group on four of the five ANAM subtests. Participants were tested 30-times over the course of 4 days (i.e., six-times on day 1, eight-times per day for the next 3 days) to attempt to replicate previous findings that examinees with mTBI had larger variability on measures of RT and response speed over multiple assessment sessions [55–57]. Their findings also revealed control participants demonstrated less variability and a practice effect over time, while the mTBI group's performance was more variable and actually declined across repeated test sessions.

In their assessment of a mixed clinical sample, Woodhouse *et al*. [21] used logistic regression to determine the classification accuracy of ANAM to predict RBANS scores that were ≤15th percentile (i.e., ‘impaired’). All seven subtests generated significant differences among the groups of healthy and impaired individuals. This model indicates that ANAM TP scores can predict impairment status with high sensitivity and specificity, suggesting that ANAM is capable of classifying impairment similarly to the RBANS. However, the RBANS is typically used in the assessment of age-related cognitive decline, and therefore may not be the most suitable assessment for postconcussion evaluation. Kelly *et al*. [24] found that, in baseline and intergroup comparisons among concussed and healthy soldiers deployed to combat environments, the best area under the curve (AUC), which is an indicator of discriminant ability (i.e., differentiate between those with mTBI and controls), came from Simple Reaction Time (SRT) TP scores. The data suggest that this distinction may be the most accurate within 72 h of injury. Some other score combinations improved ANAM's discriminant ability (e.g., SRT + Procedural Reaction Time [PRO] for normative comparisons, SRT + MTH + Matching to Sample [M2S] for baseline comparisons), though not drastically so. These results possibly provide support for using only RT- and PRO-based tasks in a potentially shorter ANAM battery. Coldren *et al*. [25] also sought to evaluate the diagnostic capability of ANAM in the combat environment and found that, in comparison to controls and baseline scores, the mTBI group demonstrated lower scores with statistically significant differences on five of the six ANAM subtests scores at ≤3 days postinjury. However, only minimal differences were found at the 5 and 10+ days intervals.

Norris *et al*. [26] found that ANAM assessments at 3- and 5-day postinjury may demonstrate prognostic utility. In their study, those soldiers who performed at or lower than 25% needed 19 days to recover and be cleared to return to duty (RTD), while those who performed in the top 25% were able to RTD in just 7 days after injury, with the largest effect sizes seen for the SRT2 subtest. Results from another study suggest that the use of ANAM as a diagnostic tool for concussion may be limited. In a sample of college football athletes, few examinees with concussion were consistently classified as impaired across ANAM and a traditional NP battery [27]. In this study, ANAM had high specificity but low sensitivity; however, when combined with the Sensory Organization Test and symptom measures, the sensitivity improved, albeit only slightly. These results indicating low sensitivity raise questions about the isolated use of ANAM or any other concussion test (Sensory Organization Test or Graded Symptom Checklist) for clinical decision-making.

Finally and most recently, Nelson *et al*. [28] prospectively compared three NCATs, including ANAM, in groups of concussed and healthy athletes at 1, 8, 15 and 45 days following injury, with similar intervals for matched controls. At 1-day postinjury, AUC was fair and scores from six of the seven subtests as well as the composite score were significantly different from the control group. At days 8 and 15 postinjury, only one subtest (M2S) showed significant differences, and there were minimal significant differences at 45-day postinjury. The authors concluded that ANAM has limited clinical utility after 8 days following mTBI.

### Summary

When evaluating the existing literature on ANAM according to the Randolph criteria [7], it does not appear that these criteria have been sufficiently addressed. Correlations with traditional NP tests are generally moderate at best, though often weaker. Moreover, the stronger correlations are not consistently between tests that purport to measure the same cognitive construct. The scores that seemed to be the most robust from the ANAM were MTH and those that are primarily RT based, often most strongly correlated with traditional tests of motor and processing speed. Therefore, construct validity as measured by correlation analyses is questionable at best. However, there are indications from regression analyses and PCA that similar cognitive constructs are being measured by ANAM and traditional NP tests, though perhaps in a slightly different manner.

The results from the existing literature also suggest that ANAM has questionable sensitivity to the effects of concussion, especially if testing is completed more than a week from injury. While the mTBI groups often displayed worse performance, more variability in performance or a lack of practice effects as compared with controls, the diagnostic utility of these differences is currently unconvincing. Though specificity was often high and approaching clinically meaningful levels, sensitivity was generally lower than desired. However, there are indications that identifying cognitive impairment rather than mTBI status may be more meaningful and yield better diagnostic accuracy. This approach was recommended by Iverson and Schatz [16] and may be the best approach to addressing Randolph *et al*.'s [7] second criterion evaluating the sensitivity to the effects of concussion.

## CNS-Vital Signs

### Comparisons to traditional NP tests

There is not a large body of published literature regarding the validity of CNS-VS. The correlational studies suggest some degree of relatedness between CNS-VS and traditional NP tests, although no consistently clear patterns have been determined (see Table 3 for details on the methodology and results of these investigations). Gualtieri and Johnson [29] found significant correlations between CNS-VS and a traditional NP test battery in groups of healthy controls and patients with various neuropsychiatric disorders, including postconcussion syndrome (PCS) and severe brain injury. CNS-VS Symbol Digit Coding and WAIS Digit Symbol Coding subtest scores were identified as the strongest correlated scores, providing some evidence of convergent validity. Another study [30] evaluating scores in a sample of examinees 6–8 weeks removed from concussion generated significant, though modest, correlations between CNS-VS and the traditional NP tests, with the strongest correlation between CNS-VS Psychomotor Speed Standard Score and the Neuropsychological Assessment Battery Memory index Standard Score. Gualtieri and Hervey [31] found that overall, correlations among the CNS-VS and traditional battery were weak to moderate (-0.33 to 0.59) in a sample of psychiatric patients. WAIS-III and -IV Full Scale Intelligence Quotient (FSIQ) and CNS-VS Shifting Attention Test were the most strongly correlated. The authors also conducted multiple regression analyses to further explore the relationship between CNS-VS and traditional NP tests, demonstrating that only two of the CNS-VS scores (Shifting Attention Test and Visual Memory Test) were significant predictors of FSIQ.

### Group differences

To date, there are three published studies looking at the diagnostic utility of CNS-VS with mTBI (see Table 3 for study summaries to supplement this text). Lanting *et al*. [32] administered CNS-VS to patients 6–8 weeks after sustaining either an mTBI or orthopedic injury. Though the mTBI group did have a higher proportion of scores at least one standard deviation below the mean, effect sizes were small and multivariate analysis of variance demonstrated no statistically significant differences between the two groups. Gualtieri and Johnson [33] compared healthy controls to a TBI cohort divided into four subgroups: those with PCS, those recovered from mTBI, those recovered from severe TBI and those who had not recovered from a severe TBI. Multivariate analysis of variance demonstrated statistically significant differences between the five groups in 18 of the 28 scores investigated. *Post hoc* *t*-tests clarified significant differences from healthy controls existed on all scores in both severe TBI groups and on five of six CNS-VS scores in the PCS group. There were no differences between the controls and those in the mTBI-recovered group. Receiver operating characteristic (ROC) curve analyses revealed which index scores better identified differences between the groups. A greater AUC identified those tests that could best distinguish between groups as the CNS-VS Psychomotor Speed index score, which had the greatest AUC (0.752), followed by the Neurocognitive Index (NCI; 0.747) and Cognitive Flexibility Score (0.708). Although these results indicate that the NCI may have some diagnostic capabilities, the authors question the clinical use of the NCI score, as it is currently only utilized in research settings and is not common in traditional NP assessment. Lastly, another study compared CNS-VS scores before and after deployment in active-duty service members. Though there were significant differences between examinees on pre- and postdeployment measures, there were no significant differences on CNS-VS performance [34].

### Summary

Unfortunately, the existing research suggests somewhat mixed, though largely unfavorable, results for the validity of the CNS-VS battery. Specifically, correlation analyses show no clear pattern of convergent or discriminant validity, and generally CNS-VS is at best moderately correlated with traditional NP tests. Group comparisons suggest no clear or clinically meaningful differences between groups with mTBI and control groups. However, there is a paucity of research for CNS-VS, and further investigation is required to address the Randolph criteria. Additional studies taking different approaches may yield different and more promising results. For example, more clinically meaningful differences may be evident when comparing those with acute mTBI to control groups, as the existing literature was based solely on assessments administered long after the acute postinjury timeframe. Additionally, Gualtieri and Johnson [33] found significant differences between still symptomatic groups and controls, suggesting identification of cognitive impairment or still symptomatic individuals may be more clinically meaningful in identifying someone as recently concussed or not.

## CogState

### Comparisons to traditional NP tests

Studies comparing CogState to traditional NP tests have typically focused on traditional tests of processing speed and executive functioning, generally finding some evidence for construct validity (see Table 4 for more detail associated with the findings). Makdissi *et al*. [35] reported statistically significant, though moderate at best, correlations between the CogState SRT subtest and the traditional NP tests (i.e., Digit Symbol Substitution Test and TMT-B) in samples of healthy controls and patients with acute mTBI. They found increases in variability and latency of responses in the dataset from these injured players. In a similar study [36], correlations were weak between the CogState battery's accuracy scores and the DSST and TMT scores; however, when CogState speed scores were used, there were several strong correlations, most notably between the DSST and the decision-making and working memory speed scores (-0.86 and -0.72, respectively). Schatz and Putz [37] reported moderate correlations between CogSport and a traditional NP battery, with SRT being the strongest correlated score with WAIS-R Digit Symbol Coding. In a study where healthy controls’ performance on CogState was compared with a larger battery of traditional NP tests, Maruff *et al*. [38] reported moderate-to-strong correlations between the various CogState domains (processing speed, attention, working memory and learning) and the traditional NP tests measuring similar constructs, suggesting support for the construct validity of CogState.

### Group differences

There have been four studies published to date looking at the difference in performance on CogState between healthy controls and mTBI patients, with mixed results (see Table 4 for accompanying details on the methodology and results). In one of the earlier studies [35], traditional NP scores did not significantly change from baseline in either the concussed or nonconcussed samples of football players, though the concussed group did demonstrate a significant (36%) decline in performance on the CogState SRT task. *Post hoc* *t*-tests demonstrated that the control and concussed groups’ SRT variability were not statistically different at baseline, but the concussed groups had significantly more RT variability at follow-up. Similarly, a prospective study of cognitive functioning following concussion in football players found that the symptomatic group of patients who sustained a concussion demonstrated a significant decline in CogState performance and no change in traditional NP test scores, while the controls and asymptomatic concussion groups mostly improved in their performance in both CogState and the traditional NP battery [39]. Maruff *et al*. [38] found evidence of criterion validity by administering CogState to three groups of examinees with cognitive impairment (mTBI, schizophrenia and AIDS Dementia Complex), and three groups of demographically matched controls. Of interest to this review, the mTBI group was significantly different from the control group, with large effect sizes observed on the OCL/learning task. In addition, the authors used a measurement called the nonoverlap statistic (non-OL%) to identify the percentage of each group's data distributions that do not overlap. Using this metric, they found that each of the CogState subtests was able to identify between 53 and 78% of the impairment unique to the mTBI group (p < 0.0001). Utilizing both baseline and normative reference groups, Louey *et al*. [40] have also provided evidence that CogState can be used to detect concussion-related cognitive impairment. The authors found that the baseline method demonstrated a higher sensitivity and comparable specificity to the normative method, and even after taking into account baseline performances, the concussed group showed performance declines. The baseline and normative methods could be used to correctly classify individuals as cognitively impaired up to an accuracy of 87.9 and 89.3%, respectively.

However, in two of the studies to date, comparisons among healthy and impaired individuals provided less convincing support of CogState's criterion validity, as CogState either only moderately improved classification between groups or, similar to ANAM, could do so only at earlier postinjury time points. Gardner *et al*. [41] administered CogState alongside ImPACT and WAIS-III to rugby players with or without acute mTBI. They observed statistically significant differences between the groups on four of the five CogState subtest scores. However, logistic regression demonstrated that CogState scores only minimally improved classification accuracy above what demographics predicted when added to the regression model. In their prospective study (previously mentioned in the ANAM review), Nelson *et al*. [28] found that at 1-day postinjury, all of CogState's subtests were significantly different from the control group. There were two subtests (Attention Speed and Learning Speed) that also demonstrated statistically significant differences at 8-day postinjury, though with small effect sizes. The ROC analyses revealed that only the CogState subtests administered at 1-day postinjury demonstrated significant AUC, suggesting that at the later time points, CogState subtests likely do not have diagnostic utility.

### Summary

Though correlations between CogState and traditional NP tests have been wide ranging, there is some evidence for convergent validity, with a general pattern of tests supposedly measuring similar cognitive constructs being more strongly correlated than dissimilar measures. Investigations of the clinical utility of CogState with concussion have had mixed results. Some tests have demonstrated the ability for CogState to distinguish between those with concussion and controls, even outside of the 7-day window (e.g., 1 month). In fact, one study suggested CogState may have had more clinical utility in postconcussion assessments than traditional NP tests [39]. Also, CogState was able to correctly classify over 88% of individuals as concussed or not by comparing scores to both normative databases and baseline performance [40].

Similar to research with other NCATs, the clinical utility of CogState may be increased by identifying individuals who are symptomatic after injury, rather than just comparing those with concussion (and possibly asymptomatic) to healthy controls. However, other studies have found that CogState's ability to detect postconcussive symptoms may be limited outside of the acute stage of injury (e.g., beyond 7 days), and even in the acute stage it may not provide much information beyond demographics. Overall, though the Randolph criteria have been largely addressed by the existing research, there is inconclusive support for meeting those criteria and additional research with CogState is warranted. The study samples and traditional NP test batteries used in the existing studies have been fairly narrow. Additionally, the wide range of correlations between CogState and traditional NP tests warrants regression and PCA to determine the extent to which CogState is measuring similar cognitive constructs to traditional NP tests. Also, additional studies investigating the clinical utility of CogState to detect cognitive impairment, both in and beyond the acute injury phase, are necessary.

## ImPACT

### Comparisons to traditional NP tests

Alsalaheen *et al*. [15] conducted a comprehensive and systematic review of the validity of ImPACT, and the intention is not to repeat their work. The reader is encouraged to consult their review for a comprehensive summary of ImPACT literature to date. Alsalaheen *et al*. [15] concluded that there is strong evidence for convergent validity of ImPACT though weak or inconclusive evidence for discriminant validity, criterion validity or diagnostic accuracy and utility. This would suggest inconclusive support for meeting the Randolph criteria [7]. Below, we highlight several studies investigating convergent and criterion validity, as well as diagnostic utility in mTBI cases (see Table 5 for study summaries to accompany the findings described below).

Iverson *et al*. [42] compared ImPACT results with those of a paper and pencil test commonly used as a measure of attention and processing speed (Symbol Digit Modalities Test [SDMT]) in a cohort of young athletes. The strongest correlations with SDMT were ImPACT's Processing Speed and RT composite scores. Exploratory factor analysis (EFA) uncovered a two-factor solution of speed/RT and memory, suggesting ImPACT is measuring similar cognitive constructs as SDMT. Schatz and Putz [37] found moderate correlations among ImPACT and a traditional NP battery in a group of healthy controls, with the strongest correlation being ImPACT Choice RT score and Trails A. Similarly, Maerlender *et al*. [43] found that ImPACT was moderately correlated with tests of similar cognitive domains. Canonical correlation analyses indicated that two of the five canonical dimensions were statistically significant, with coefficients of 0.801 and 0.729, confirming that the two batteries generally measure similar cognitive constructs. However, a follow-up study by the same authors [44] re-analyzed the 2010 dataset to specifically evaluate the discriminant validity of ImPACT as compared with traditional NP tests. The results indicated that while the traditional battery demonstrated evidence of discriminant validity (i.e., all domains’ p-values > 0.05 except RT), ImPACT did not discriminate between measures of different cognitive skills. Specifically, three of the four domain scores were strongly correlated with expectedly different traditional NP measures.

Allen and Gfeller [45] compared performance measures of ImPACT to those of the NFL NP battery, which consists of the Hopkins Verbal Learning Test-Revised, Brief Visual Memory Test-Revised, TMT, Controlled Oral Word Association Test and three subtests from the WAIS-III, in a sample of healthy controls. Correlations were moderate at best, with the strongest correlation between WAIS-III Coding and ImPACT's Visual Motor Speed Composite. Solomon and Kuhn [46] examined the relationship between performance on the Wonderlic and ImPACT in 226 NFL draft picks with and without a history of concussion. Concussion history did not have a significant effect on performance on either of the tests. Correlations between the batteries were weak to moderate, with Visual Motor Speed being the most strongly correlated with Wonderlic performance.

### Group differences

Studies to date comparing ImPACT to a variety of traditional NP tests and among many different patient populations have corroborated that ImPACT may be useful as a diagnostic tool postconcussion, and perhaps even the most sensitive of the four NCATs described in this review (see Table 5 for supporting summaries of each study). Van Kampen *et al*. [47] compared the ImPACT performance of college athletes with acute concussion to matched controls, also utilizing preseason baseline assessment scores. RCI scores defining abnormal performance indicated that 83% of the participants in the mTBI group performed abnormally lower than their baseline. When cognitive data were combined with symptom questionnaires, 93% were categorized as abnormal. However, 30% of the control group also generated abnormal ImPACT test data or self-reported symptoms. Broglio *et al*. [48] reported that groups of students with and without acute mTBI differed on all indices except Impulse Control. Furthermore, the ImPACT battery demonstrated better sensitivity to mTBI (79.2%) than a traditional NP battery (43.5%). Similarly, in a study of recently concussed college athletes, Covassin *et al*. [49] found that there were significant differences in Verbal Memory and RT based on whether participants had a history of prior concussion (i.e., those with a prior concussion performed worse) at both 1- and 5-day postinjury. Schatz *et al*. [50] also observed that ImPACT classified a group of recently concussed high school athletes with a sensitivity/specificity of 81.9/89.4. Schatz and Maerlender [51] performed factor analyses using existing ImPACT datasets, which included 21,537 baselines and 560 postinjury assessments. They identified two primary cognitive factors, memory (comprised of Verbal and Visual Memory domains) and speed (comprised of Visual Motor Speed and RT domains), that accurately classified individuals as concussed or not concussed, with a sensitivity/specificity of 89/70.

However, there has not been universal evidence that ImPACT adequately differentiates between healthy controls and recently concussed individuals. As previously mentioned, Gardner *et al*. [41] administered ImPACT, CogSport and WAIS-III to professional rugby players with acute mTBI and to matched controls. They found statistically significant differences between the groups on only one of the four ImPACT composite scores (Visual Motor Speed). Logistic regression demonstrated that ImPACT scores were unable to distinguish between the injured and control groups beyond demographic variables, as ImPACT scores only added 3.5% improvement in accuracy to the overall classification model. ROC curve analyses demonstrated modest sensitivity and specificity for the ImPACT composite score.

There is additional support for the clinical utility of ImPACT from studies investigating the test's ability to distinguish between symptomatic and asymptomatic mTBI patients. Schatz and Sandel [52] administered ImPACT to groups of high school and college athletes with acute mTBI (symptomatic and asymptomatic) within 72 h of injury. The data were compared with demographically matched controls with pre- and postseason assessments. ImPACT data demonstrated the ability to detect differences between the groups (sensitivity/specificity of 91.4/69.1 and 94.6/97.3 for the symptomatic and asymptomatic groups, respectively). In the prospective NCAT comparison by Nelson *et al*. previously described [28], all ImPACT composite scores were significantly different at 1 day following injury. However, there was only one score that was significantly different, and with a small effect size, after this timeframe (day 8, Verbal Memory, *d* = -0.40). Likewise, AUC for the composite scores were fair at the 1-day postinjury assessment [0.70,0.71] though poor (<0.69) for the other timeframes. However, of the three NCATs evaluated, ImPACT demonstrated the highest percentage of test scores that significantly declined from baseline to 1-day postinjury according to the RCI criteria (67.8% for both symptomatic and asymptomatic concussed populations), although the test also had a slightly higher false-positive rate than ANAM and CogState in the same 24-h period (29.6% compared with 25.0 and 22.0%, respectively). When examinees were dichotomized as symptomatic or not, ImPACT also demonstrated the largest percentage of patients with a significant decline from baseline performance (53.8% at 1-day postinjury).

### Summary

ImPACT is the most widely studied of the NCATs, and as such the Randolph criteria [7] have been thoroughly addressed through the existing body of research. Though the Randolph criteria have been satisfied to a degree, as Alsalaheen *et al*. [15] concluded that there are mixed results regarding the overall validity of ImPACT. Specifically, there appears to be solid evidence that ImPACT has adequate relatedness with traditional NP tests, especially those purported to measure similar cognitive constructs. More advanced statistical approaches suggest there is also evidence that ImPACT is measuring similar cognitive constructs to traditional NP testing. However, there is not a clear pattern of weaker relationships between tests of dissimilar cognitive constructs, calling into question the discriminant validity of ImPACT. ImPACT's tests of RT and processing speed, especially Visual Motor Speed, seem to have the most robust relationships with traditional NP tests. And with regard to identifying postconcussion issues, ImPACT does show the ability to distinguish between concussed and noninjured individuals during the early stages postinjury. And though sensitivity is generally better than specificity, there were some studies that found comparable sensitivity and specificity, both of which approached desired levels for clinical decision-making. However, after the early postinjury stages, and certainly outside of 7 days, the clinical utility of ImPACT for postconcussion assessments appears limited. Improved clinical utility may be demonstrated if identification of symptomatic individuals postinjury is the focus, rather than identifying individuals as concussed or not.

## Discussion

The goal of this review was to provide a summary of literature regarding the validity on four commonly used and studied NCATs: ANAM, CNS-VS, CogState and ImPACT. The literature was viewed through the lens of Randolph *et al*.'s criteria presented in their 2005 [7] literature review of NP testing after SRC (Box 1). NCATs are becoming the standard of care for mTBI screening in athletic and military deployment settings given the improvement in efficiency and feasibility of test administration over their traditional NP counterparts. However, it is clear from the above summary of the literature to date that there has yet to be definitive evidence in support of the validity of any of the four NCATs, per Randolph's validity-related criteria (i.e., criteria two through five).

Currently, the body of literature suggests mixed results regarding NCATs’ validity. Specifically, there is evidence that NCATs measure similar cognitive constructs as traditional NP tests (i.e., Randolph's 3rd criterion). And there is some support that NCATs, or at least components of each NCAT, can distinguish between individuals with acute concussion and healthy controls, or between still symptomatic individuals and individuals who are symptom free (i.e., Randolph's 2nd criterion and 5th criterion). However, there is little to no evidence for discriminant validity as compared with traditional NP tests, and inconsistent evidence for the clinical utility of NCATs for identifying concussion-related problems, especially beyond the first 7-day postinjury and when the tests are used in isolation. We did not review the literature regarding Randolph's 1st criterion, related to test–retest reliability, as this was beyond the scope of the paper. With regard to Randolph's 4th criterion, establishing RCIs and probability-based algorithms for clinical use is dependent on well-established test–retest reliability and well-defined constructs of the tests. That is, we need to know what the test is measuring, how it is measuring it and how consistently it does so before we can calculate them. As such, additional research will be needed before any of the NCATs fully satisfy the criteria for validity and ultimately for clinical utility.

Although there is not consistent evidence regarding the validity and clinical utility of NCATs, and the criteria presented by Randolph *et al*. [7] have not been sufficiently addressed, there is evidence suggesting that NCATs are of potential benefit in postconcussion assessments. It may be that the tests are fundamentally different than traditional NP tests, and therefore using traditional NP tests as a point of comparison, or using traditional psychometric approaches to defining validity creates a logical fallacy of false analogy or an ‘apples to oranges’ comparison. That is, perhaps NCATs should not be faulted for not being a good proxy for traditional NP tests, but rather should be investigated as an altogether different assessment tool. Therefore, we explore future directions for this field of research through the lens of the Randolph criteria.

Studies should seek to address Randolph's 2nd and 5th criteria by designing studies that “establish the ability to identify cognitive impairment after concussion and distinguish between individuals who are symptomatic and those who are asymptomatic post-injury”. This approach shifts away from a group-based approach (e.g., mTBI vs controls) that has dominated the literature to date, focusing on cognitive impairment and symptom-driven approaches, while allowing for a wider range of methodology in future studies. There were several studies identified in this review that demonstrated NCATs consistently found more clinically meaningful differences between symptomatic versus asymptomatic groups, and that asymptomatic individuals often performed like healthy controls [28,39,52]. Similar future research may prove more valuable in elucidating the clinical utility of these tests.

This impairment and symptom-based approach is also consistent with the recommendation of Iverson and Schatz [16] to specifically investigate cognitive impairment rather than mTBI status. They go further and describe new approaches to identifying cognitive impairment, such as taking a base rate approach and categorizing performance based on the total number of low scores across a battery. Determining clinically meaningful definitions of cognitive impairment, and then establishing the NCATs’ sensitivity and specificity in classifying individuals with concussion as cognitively impaired, will be key to further establishing the validity, and ultimately, the clinical utility of NCATs, especially with regard to informing return to play and RTD decisions.

Randolph's 3rd criterion may be addressed by studies seeking to “determine what cognitive constructs NCATs are measuring, and if those constructs, and the manner in which they are measured, are clinically meaningful”. This direction is suggested in light of the evidence that the standard statistical approaches of assessing validity have yielded at best moderate convergent validity, poor discriminant validity and inconsistent evidence that NCAT scores predict traditional NP scores. However, alternative statistical approaches, such as PCA and EFA, have suggested NCATs are measuring similar cognitive constructs, though perhaps in different ways. Specifically, it may be that the names given to NCAT subtests and index scores may not accurately reflect the actual cognitive construct being measured. Therefore, statistically guided comparisons, rather than those guided by nomenclature, could yield better evidence for convergent and discriminant validity.

We also recommend a shift away from ‘standard psychometric procedures’ since this often relies on comparisons to a gold standard, such as traditional NP tests. However, there is mixed evidence for the utility of traditional NP tests for use in postconcussion assessments, especially outside of the acute injury phase [24]. NCATs are typically presented as potential proxies for traditional NP tests, and as such, validity is often evaluated by direct comparisons between supposedly comparable tests. However, adapting pencil and paper tests to a technological interface can fundamentally change the test. Some have suggested that an NCAT's ability to precisely measure RT may be an advantage over traditional NP tests in detecting subtle cognitive declines after concussion [5]. In fact, RT and processing speed scores are often the most robust in studies predicting concussion status or cognitive impairment. Also, several studies have identified alternative scores or interpretative methods that may provide more clinical utility than the standard scores currently provided. For example, RT variability and lack of practice effects may be more sensitive to concussion-related effects [22–23,35,58–60]. Thus, the potential technological advantages provided by NCATs warrant closer investigation. A caveat, however, as others have identified sources of error that are introduced into test scores due to the use of technology. This includes a participant's familiarity with using a computer [61] to hardware and software configurations [4,62–63]. The literature is limited in identifying how technology can affect the measurement of performance, and this will be important to clarify in future studies.

There are several other considerations with regard to the manner in which NCATs assess cognitive functioning, and the subsequent impact on clinical utility. First, though comparisons to baseline assessments have routinely been used with NCATs, and can be helpful in the context of cognitive changes in examinees with pre-existing unique cognitive abilities (i.e., upper or lower 20th percentile, ADHD or LD), the use of baseline testing does not appear to be necessary for determining cognitive deficits following concussion [64,65]. Research should focus on the ability for baseline assessments, normative comparisons or some combination of the two to accurately and adequately identify symptomatic individuals. Also, the use of group versus individual settings during test administration should be considered, as there is mixed evidence regarding the potential impact a group versus individual test setting has on test scores [66,67]. The different administration settings could potentially impact the findings as NCATs are often administered in group settings either preseason in athletics or predeployment in the military, and then individually postinjury. Additionally, the environment in which NCATs are often desired to be administered, such as athletic sidelines or combat zones, is an important consideration as much of the research takes place in highly controlled settings. The clinical utility of NCATs in such austere environments warrants further investigation [68,69].

## Conclusion & future perspective

Though the body of literature regarding the validity of the four NCATs discussed in this review has been steadily growing, there appears to be insufficient evidence suggesting that these tools are adequate proxies for traditional NP tests and have limited clinical utility in postconcussion assessments. However, by investigating NCATs with the same methodology used to investigate traditional NP tests, these tests may have been set up for failure. Using the 2005 Randolph criteria, we have provided additional and alternative ways forward for investigating the validity and utility of NCATs that are better suited for the intended clinical use and design of these tests. Future efforts are encouraged to focus on cognitive impairment (e.g., symptomatic vs asymptomatic) rather than group status (e.g., concussed vs controls), the ability to inform return to play and RTD decisions, and utilization of alternative and novel statistical approaches (e.g., RT variability, base rate analyses to identify impairment, etc.). Additional prospective comparisons of multiple NCATs in differing study samples, similar to the one conducted by Nelson *et al*. [28] are also warranted. NCATs have the potential to fundamentally change the nature of care following mTBI. However, until their clinical utility can be further established and clarified, they should be used with caution and at most as screening tools in combination with multifaceted assessments.

**Box 1.**  **‘Randolph criteria’ for proposed neuropsychological batteries.**

**Criterion & description**

- Establishing test–retest reliability over time intervals that are practical for this clinical purpose
- Demonstrating, through a prospective controlled study, that the battery is sensitive in detecting the effects of concussion
- Establishing validity for any novel test battery, through standard psychometric procedures employed to determine which neurocognitive abilities a new NP test is measuring
- Deriving reliable change scores, with a probability-based classification algorithm for deciding that a decline of a certain magnitude is attributable to the effects of concussion, rather than random test variance
- Demonstrating that the proposed battery is capable do detecting cognitive impairment once subjective symptoms have resolved

Note: These are criteria set forth by Randolph *et al*. [7] for both traditional and computerized NP batteries. Randolph *et al*. proposed that NP tests should first meet these criteria prior to their consideration as part of routine standard of care for sport-related concussion.NP: Neuropsychological.

**Box 2.**  **Evidence of validity.**

**Content-related**

- The relevance of the test items to the construct that is to be measured
- Evaluated by the subjective agreement among subject matter experts, such as neuropsychologists, that the test items are relevant and appropriate for the test purpose

**Construct-related**

- The extent to which the measure represents the basic theoretical construct
- Evaluated with correlations, regression or factor analysis between a domain of interest and other well-established, ‘gold-standard’ measures

**Criterion-related**

- The relatedness of the measure to a specified criterion, such as a condition of interest or outcome (e.g., mTBI)
- Evaluated by the group differences, accuracy of diagnosis or identification of a specific condition of interest

It is important to note that there can be some overlap in these different types of validity, as they are not meant to conceptually represent mutually exclusive subcategories of validity, but rather describe the various ways in which validity can manifest [12].mTBI: Mild traumatic brain injury.

**Executive summary**

**Background**

- The assessment of cognitive functioning has been identified as an important aspect in the management of concussion.
- Due to logistical advantages over traditional neuropsychological (NP) tests, computerized Neurocognitive Assessment Tools (NCATs) have gained popularity in athletic and military settings.
- However, the psychometric properties, especially validity, and clinical utility of NCATs have yet to be consistently established.

**Neurocognitive assessment tools**

- Automated Neuropsychological Assessment Metric is commonly used to assess cognitive functioning in US Military Service Members.
- CNS-Vital Signs is commonly used in psychiatric and neurological clinical trials.
- CogState/Axon/CogSport is commonly used in Australian athletics.
- Immediate Post-Acute Concussion Test (ImPACT) is the most widely used NCAT in US athletics. It has the US FDA approval for postconcussion assessments.

**Existing evidence for validity & clinical utility**

- Automated Neuropsychological Assessment Metric related best with traditional NP tests of processing speed, with evidence of moderate sensitivity/specificity for concussion or postconcussive symptoms during the acute injury period.
- CNS-Vital Signs had the least amount of validity-related research, with findings revealing at best moderate correlations with traditional NP tests and no clear evidence for clinically meaningful differences between concussed and controls, though data were from the postacute injury timeframe.
- CogState demonstrated some evidence of validity with several moderate to strong correlations to traditional NP measures and the ability to detect concussion-related cognitive decline during the acute injury period. However, research has had a narrow focus on primarily reaction-based scores and with Australian athletes.
- ImPACT is the best studied of the NCATs, with research indicating mixed results regarding validity. It does appear ImPACT is measuring similar cognitive constructs as traditional NP tests, with some evidence for detecting concussion-related cognitive decline during the acute injury period at levels approaching those desired for clinical decision-making.

**Future perspective**

- Additional investigation of the validity and clinical utility of NCATs is warranted, with future efforts encouraged to focus on cognitive impairment (e.g., symptomatic vs asymptomatic) rather than group status (e.g., concussed vs controls), the ability to inform return to play and return to duty decisions and novel statistical approaches (e.g., reaction time variability and base rate analyses to identify impairment).
- Additional prospective comparisons of multiple NCATs in differing study samples, similar to the one conducted by Nelson *et al*. (2016) are also warranted.
